# PRESIDENTIAL SYMPOSIUM: FUNDAMENTAL APPROACHES TO HEALTHY AGING

**DOI:** 10.1093/geroni/igae098.0579

**Published:** 2024-12-31

**Authors:** Nathan LeBrasseur

**Affiliations:** Chair

## Abstract

The major threats to a long and healthy lifespan in humans include heart disease, stroke, cancer, diabetes, and Alzheimer’s disease; conditions for which the risk rises exponentially with advancing age. Healthy lifestyle choices are currently the most effective strategies to delay if not prevent the onset and progression of each of these distinct conditions, which suggests that they counter the fundamental biology of aging. In this Presidential Symposium, thought leaders in nutrition (Dr. Peterson), exercise (Dr. Fielding), sleep (Dr. Carroll), and circadian rhythms (Dr. Panda) will share their insights into how lifestyle choices influence biological mechanisms and, in turn, the clinical manifestations of aging.

